# Editorial: Integrative biophysical models to uncover fundamental processes in plant growth, development, and physiology

**DOI:** 10.3389/fpls.2026.1837360

**Published:** 2026-04-14

**Authors:** Bijayalaxmi Mohanty, Ingo Dreyer

**Affiliations:** 1 NUS Environmental Research Institute, National University of Singapore, Singapore, Singapore; 2 Electrical Signaling in Plants (ESP) Laboratory – Center of Bioinformatics, Simulation and Modeling (CBSM), Faculty of Engineering, Universidad de Talca, Talca, Chile

**Keywords:** biophysics, interdisciplinarity, modeling, multidisciplinary, plant physiology

Plant growth and development are governed by a multitude of complex interactions between genetic, biochemical, and environmental factors, each of which influences the structure and function of living systems. Understanding these fundamental processes is crucial for advancing our knowledge of plant biology and improving agricultural practices. However, traditional wet-laboratory approaches often fail to capture the dynamic and integrative nature of these processes. The application of biophysical models provides a powerful framework for bridging gaps in understanding and knowledge and enables us to explore how physical and chemical forces, molecular mechanisms, and environmental factors interact to govern plant behavior. In fact, in the last years, biophysical modeling has become an increasingly important tool for deciphering fundamental processes in plants at various organizational levels. From the molecular dynamics of individual membrane proteins to water and substance transport throughout the entire organism, mathematical and physically based models enable a quantitative understanding of biological mechanisms.

To highlight the broad applicability of such modeling approaches, we have compiled a collection of research and review articles on the topic of “integrative biophysical models” that address key questions in plant biology. A total of 13 articles by 74 authors from 10 different countries cover a broad spectrum of the topic. They address, for example, (i) a broad spectrum of modeling approaches for predicting plant growth and development, including the effects of thinning on growth and yield of forest trees, spatiotemporal water demand, variations in branch growth, wheat growth, fruit nutritional status, leaf phenotype, leaf vein patterns, and secondary metabolites; (ii) challenges in modeling the effects of abiotic stress factors, such as frost, heat stress and water flow stress, on plant development and yield, (iii) the use of models to mitigate the emission of greenhouse gases from agriculture, as well as (iv) the application of modeling in modern agricultural technology for food production, particularly smart irrigation management techniques.

## Biophysical modelling to predict plant growth and development

The article by Zhao et al. uses the example of *Pinus koraiensis* in northeastern China to demonstrate how important—compared to individual effects—the interactions of various parameters such as branch age, tree size and height growth, competition, and climate are for improving the branch growth model. They were able to show that the extent of the effects of climate and competition depends on the social status of the trees. Dominant and intermediate individuals are more sensitive to competition than suppressed individuals. The model revealed that desirable branch growth depends on the influence of climate. Their study underscored the importance of understanding the various causes of fluctuations in branch growth, as this could contribute to the development of sustainable management strategies in response to the uncertainties and complexity of climate change.

Secondary metabolites in plants are crucial for survival, defense, signaling, and responses to environmental stresses (Jan et al., 2021). In their article, Devadze et al. successfully predicted the content of secondary metabolites using data-driven methods by integrating and analyzing time-series data from greenhouse measurements. The content of secondary metabolites in hydroponically grown greenhouse tomatoes was forecasted, taking into account all climatic changes as well as photosynthesis and transpiration rates throughout the entire growing season. They demonstrated that the optimal accumulation of individual phenolic compounds and carotenoids in tomatoes depends on environmental factors such as radiation, temperature, relative humidity, and CO2 concentration, as well as plant transpiration and photosynthesis during the growing season. Another objective was to achieve the maximum content of secondary metabolites in tomatoes in order to obtain an optimal amount of health-promoting phytochemicals for the benefit of human health.

Holloway et al. (2025) reformulated the hypothesis regarding the regulation of leaf vein patterning and channeling by PINs (membrane-bound proteins involved in auxin efflux from cells) and plasmodesmata (PD) in relation to the formation of auxin-provascular strands during early leaf development. Their model combines the dynamics of both PD and PIN and assumes that the entire channeling mechanism arises from the contribution of all components. Furthermore, compared to models that consider only PIN, the model yields better results regarding the number, orientation, and extent of the veins under conditions of reduced transport by PINs.

Biophysical models also serve as a powerful tool for evaluating thinning performance in Chinese fir (*Cunninghamia lanceolata*) plantations, which affects growth and yield. In subtropical China, the Chinese fir is an important source of timber. To evaluate this thinning performance, Jiang et al. compared two growth models: the “Compatible” model and the “Annual” model (periodic-annual). The authors demonstrated that annual growth models perform best over short time horizons, while compatible growth models perform better over medium to long time horizons.

Ru et al. developed a phenological model to assess the spatial and temporal water requirements for effective water management and sustainable apple production in China’s major apple-growing regions. They found that the water requirements of apple trees vary significantly depending on the phenological stage and region. Therefore, they emphasized the importance of optimizing irrigation practices to promote sustainable apple production in China.

Plant phenotypes such as shape, size, texture, color, and anatomical structures are important indicators of plant growth status (Zhao et al., 2025). In the frame of this Research Topic, Wang et al. developed an algorithm for skeletonization by connecting spontaneous key points based on random points for the phenotypic extraction of leafy plants. Their algorithm can spontaneously detect key points and extract skeletons according to plant morphology. Therefore, it can be effectively used for accurate phenotypic extraction in leafy plants with complex morphologies.

In addition, we have a research article by Yu et al. on the total phosphorus content of the leaves. The nutrient status of the fruit depends mainly on the phosphorus content of the leaves (Bai et al., 2021). On this base, Yu et al. systematically predicted the total phosphorus content (LTP) of Korla fragrant pear leaves using near-infrared spectroscopy. This model prediction, based on the specificity of the growth stage, also significantly improved the accuracy and applicability of the model.

Finally, we would like to draw attention to a review article by Dusfour-Castan et al. on wheat growth models that take into account physiological trade-offs between growth, defense, and reproduction, as well as hormonal signaling and gene regulatory networks. Such methods can improve and support experimental design for physiological questions such as plant growth and resistance.

## Challenges in modelling the impact of abiotic stress on crop development and yield

Increasing frost and heat stress due to the uncertainty of climate change are key limiting factors for plant growth and development and ultimately affect crop yield (Rezaei et al., 2023). The review article by Richetti et al. focuses on the effectiveness of yield simulation models for the yield of cool-season annual cereal crops. The authors argue that modeling approaches are inadequate due to six major challenges and that model predictions can be improved through the refined use of sub-daily climate data, the incorporation of physiological damage mechanisms, and enhanced crop- and genotype-specific parameterization.

The research article by Hara et al. examined how flow stress inside and outside river bends differentially affects the morphological, anatomical, and mechanical properties of populations of *Osmunda x intermedia* (*Osmundaceae*) growing inside and outside river bends, respectively. Their study showed that, for *O. x intermedia*, internal anatomical and mechanical changes are more critical for adaptation to different types of flow stress than external morphology.

Climate change due to rising greenhouse gas emissions from Agriculture, Forestry, and Other Land-Use sectors (AFOLU) is one of the most significant global environmental challenges (Bumyut et al., 2026). Within the Research Topic, Kwak et al. presented a systematic review of allometric equations for orchard and vineyard trees using five large databases (2008–2024), following the PRISMA guidelines to improve AFOLU-based climate change mitigation. Their research shows that these perennial fruit tree systems represent an important step in carbon accounting frameworks to support climate-friendly agriculture and can contribute to achieving national and global carbon neutrality goals.

## Application of modelling to modern agricultural technology for food production

Modern agricultural technologies are crucial for global food security and environmental sustainability. As part of this Research Topic, Tang et al. have introduced a smart irrigation management technique specifically designed for greenhouse systems, which offers great potential for sustainable agricultural practices through the use of reinforcement learning. They developed the ENPPO algorithm (Enhanced Negative-incentive Proximal Policy Optimization) by incorporating real-time sensor data and historical irrigation records, thereby taking the first step toward creating a digital twin of the system. Compared to conventional methods such as PPO and TRPO, their method more accurately determined the optimal irrigation amounts for the various growth stages of different vegetable varieties. It also improved both the yield and the quality of the vegetables.

Finally, we have an excellent review article by Deb and Chakrabortty on biophysical modeling as a tool for investigating plant growth, development, physiology, and regulatory pathways. Such modelling approaches have significantly expanded our understanding of plant biology. The authors critically analyzed a wide range of computational and mathematical models, including mechanical and geometry-based models for tissue growth and morphogenesis, hydraulic and electrophysiological models for physiological transport processes, reaction-kinetic and Boolean network models for molecular and genetic regulation, as well as metabolic and constraint-based models for resource allocation. Deb and Chakrabortty proposed that many predictive tools for biotechnological applications can be developed by combining various modeling approaches, including AI-based approaches, to improve the predictive power of the models.

Overall, this Research Topic brings together research and review articles from various perspectives that explore integrative modeling approaches aimed at understanding complex physiological, biochemical, anatomical, regulatory, and environmental stress responses using a variety of predictive tools.
